# Genetic divergence among toxic and non-toxic cyanobacteria of the dry zone of Sri Lanka

**DOI:** 10.1186/s40064-016-3680-5

**Published:** 2016-11-28

**Authors:** Harshini M. Liyanage, Dhammika. N. Magana Arachchi, Naduviladath V. Chandrasekaran

**Affiliations:** 1 National Institute of Fundamental Studies (NIFS), Hantana Road, Kandy, 20000 Sri Lanka; 2 Department of Chemistry, University of Colombo, Colombo 3, 00300 Sri Lanka

**Keywords:** Cyanobacteria, Cylindrospermopsin, Microcystin, Phylogeny, 16S rRNA gene

## Abstract

Sri Lanka has rich cyanobacterial diversity, however, only few studies have been conducted to identify the potential toxin producers in water bodies used for human consumption. As the detection of cyanotoxin is vital in water quality management, a study was done by employing 16S rRNA gene to explore the genetic divergence, phylogenetic relationships and potential toxin producing cyanobacteria in reservoirs and well waters in the dry zone of Sri Lanka. Forty five, 16S rRNA gene sequences were assayed and phylogenetic tree was constructed. Among 45 isolates, 20 isolates were classified as unidentified cyanobacteria and considered as novel cyanobacterial genera. Of 25 identified isolates, seven isolates were identified up to species level. With 16S rRNA phylogeny, 20 unidentified cyanobacterial isolates were able to place on their taxonomic positions up to order level. Results revealed that water samples understudy had vast cyanobacterial diversity with potential microcystin (MC) and cylindrospermopsin (CYN) producers and eleven clusters clearly demonstrated five cyanobacterial orders with more than 90% similarity irrespective to their toxicity which showed the suitability of 16S rRNA gene for taxonomic differentiation. Sixteen isolates had the potential to produce MC and two isolates to produce CYN. Findings of the study confirm the rich cyanobacterial diversity and the divergence among the potential cyanotoxin producers in the dry zone water bodies of Sri Lanka.

**Electronic supplementary material:**

The online version of this article (doi:10.1186/s40064-016-3680-5) contains supplementary material, which is available to authorized users.

## Background

Cyanobacteria, previously known as Blue Green Algae (BGA) are oxygenic photosynthetic gram negative prokaryotes (Whitton and Potts 2000; Olson 2006). They are widespread, extremely adaptable and successful group, colonizing in diverse ecosystems (Whitton 2012). Further, they exhibit the most diverse and complex morphology among all prokaryotic groups. Their external gross appearance depends on their external environment and is often unicellular, colonial and multicellular filamentous forms, with colours ranging from dark green, blue-green, yellow, brown to black, and rarely red (Kulasooriya 2011). Traditionally, cyanobacterial classification has been based on morphological characters, which can vary in different environmental or growth conditions and even can lose during cultivation (Castenholz and Waterbury 1989; Zapomělová et al. 2008; Hasler et al. 2011). Further, certain species cannot grow in the laboratory conditions (uncultured cyanobacteria) (Ward et al. 1995) and therefore it is difficult to classify cyanobacteria into correct taxonomic groups in botanical nomenclature. However, with the advent of new molecular biological tools and along with morphology, cyanobacteria is now being classified into five orders namely; Chroococcales, Pleurocapsales, Oscillatoriales, Nostocales and Stigonematales (Castenholz and Waterbury 1989; Castenholz 1992, 2001) which are similar to the Bergey’s five subsections (I–V) described by Stanier et al. in 1978 (Stanier et al. 1978; Rippka 1988).

Phylogeny of cyanobacteria represents their evolutionary development and history with time. Molecular data have become a popular tool for phylogenetic analysis of the cyanobacteria (Kulasooriya 2011). A number of genetic marker genes have been utilized or phylogenetic analyses of cyanobacteria including 16S rRNA (Neilan et al. 1997; Wilmotte and Herdman 2001; Wu et al. 2011), the phycocyanin operon (Neilan et al. 1995, Robertson et al. 2001, Premanandh et al. 2006), randomly amplified polymorphic DNA (RAPD) PCR (Neilan 1995), the internal transcribed spacer region (ITS) of the 16S-23S rRNA (West and Adams 1997; Boyer et al. 2001), *nif* genes (Henson et al. 2004) and *rpoC* gene (Wilson et al. 2000; Wu et al. 2011). Among them, 16S rRNA gene sequence is the most widely applied strategy for assessing cyanobacterial biodiversity in nature (Ludwig and Schleifer 1994; Ercolini 2004). Additionally, for broad phylogenetic studies, sequence data from the 16S rRNA gene are the most commonly used housekeeping genetic marker due to a number of reasons. These reasons include its existence in all bacteria (often existing as a “multigene family” or as “operons”), the function of the 16S rRNA gene has remained constant over the time and therefore random sequence changes are a more accurate measure of evolution, and the 16S rRNA gene is large enough for informatics purposes **(**Patel 2001). DNA sequence information for the small subunit rRNA gene acquired from cyanobacterial culture has used to investigate the presence of cyanobacteria and their abundance in environment (Rudi et al. 2000). Furthermore, the comparative analyses of 16S rRNA gene sequences provide a novel approach to investigate the difference between strain collections and natural communities (Weller et al. 1991; Ferris et al. 1996). As sequences of 16S rRNA genes are growth independent can easily be captured by PCR from small amounts of DNA extracted from laboratory cultures or natural environments (Giovannoni 1991; Rajendhran and Gunasekaran, 2011). Cyanobacterial specific primers have been developed for the 16S rRNA gene (Nübel et al. 1997; Lepère et al. 2000; Valério et al. 2009).

Cyanobacteria are well acknowledged for their ability to produce potent toxins which have been accountable for numerous livestock and human poisonings (Chorus and Bartram 1999; Kuiper-Goodman and Fitzgerald 1999; Codd et al. 2005; Dittmann and Wiegand 2006). Among them, microcystins (MCs) and cylindrospermopsins (CYNs) are considered as most predominant and potent cyanotoxins in fresh waters that cause acute and chronic illnesses in animals and humans. They are well documented for hepatotoxicity, cytotoxicity and potential carcinogenicity, preliminarily affecting to liver and kidney functions (Chorus and Bartram 1999; Codd et al. 1999; Stewart et al. 2006).

Sri Lanka has a wide range of topographic and climatic discrepancies which leads the occurrence of diverse ecological niches providing excellent growth conditions for varied cyanobacteria. Cyanobacterial blooms have been recorded from different parts of the country (Jayatissa et al. 2006; Magana-Arachchi et al. 2009) which could be due to intensive agricultural practices (pesticides and fertilizers), domestic and industrial effluents, cattle domestication and inadequate management of watersheds (Wimalawansa and Wimalawansa 2014, 2015). It was evident that since early eighties, a dense dark green bloom of *Spirulina* sp. and *Microcystis aeruginosa* co-exists in the Beira Lake (6.9294°N, 79.85 42°E). Later, it was recorded that Beira Lake was nourished with other cyanobacterial species such as *Synechocystis*, *Lyngbya*, *Anabaena*, *Synechococcus*, *Limnothrix*, *Calothrix*, *Pseudanabaena* and *Leptolyngbya* along with potentially toxic *M. aeruginosa* (Magana-Arachchi et al. 2011). Further, *Microcystis* spp has been recorded from Kotmale reservoir (7°03′39″N, 80°35′50″E) in 1991. The bloom had a serious threat on operational activities but disappeared gradually with the influx of water during the following rainy season. The outbreak of *M. aeruginosa* in the Kotmale reservoir was an indication of Nitrogen (N) and Phosphorus (P) loading into the reservoir (Silva and Wijeyaratne 1999). Also a scum of *Anabaena and Aphanizomenon* was recorded from Parakrama Samudra reservoir (7.8996°N, 80.9705°E) in 1993 and disappeared with the release of water (Silva and Wijeyaratne 1999). Reports from Silva and Samaradiwakara (2005) showed the presence of toxigenic cyanobacteria in the Kandy Lake (7.2912°N, 80.6421°E) and their dominance in most of the reservoirs of the Mahaweli river basin (Silva and Wijeyaratne 1999). Further, Silva (2003) and Liyanage et al. (2010) recorded the occurrence of toxic *M. aeruginosa* in Kandy Lake. In the dry zone, *C. raciborskii* was recorded as the dominant cyanobacterial species in Kala wewa, Tissa wewa, Nuwara wewa and Jaya ganga in Anuradhapura District. *Aphanizomenon* spp.*, Merismopedia* spp., *Chroococcus* spp., which were comparatively moderate to low in numbers were also observed in all the four tanks along with *M. aeruginosa*, *Anabaena* spp*., Phormidium* spp., *Microcystis* spp., *Limnothrix* spp., *Pseudanabaena* spp., *Arthrospira* spp., *Gloeocapsa* spp. and *Planktothrix* spp. (Magana-Arachchi and Liyanage 2012). This *C. raciborskii* dominancy was also observed by Zoysa and Weerasinghe (2016) in Nuwara wewa and Tissa wewa reservoirs. Sethunge and Manage (2010a, b) recorded the dominancy of toxic bloom forming *M. aeruginosa* in the dry zone reservoirs in the country. Further, *C. raciborskii* was recorded from Lake Gregory (6.9563°N, 80.7803°E) which is located in the upland wet zone during the driest period in 2011 (Perera et al. 2012). Though there were number of wide spread morphological incidences of cyano-blooms recorded in Sri Lanka, the availability of molecular data for this episode is still narrow. Further, the dry zone of Sri Lanka has a vast number of reservoirs which are actively or passively serve as drinking water reservoirs providing water for urban and rural communities. Also these reservoirs are used for irrigation and other recreational activities and are also of an aesthetic value. As a developing country, Sri Lanka is gravely concerned on increasing reports of serious health problems resulting from unsafe drinking water especially in the dry zone of Sri Lanka (Dissanayake and Weerasooriya 1987; Dissanayake 1996, 2005; Rango et al. 2015). Therefore, it is important to investigate the water quality from cyanobacterial aspect that could deteriorate water quality. However, these dry zone reservoirs have not been genetically assessed either for any cyanobacterial diversity or cyanotoxin producers.

Therefore, in this study, we attempted to employ partial sequences of 16S rRNA gene to explore genetic divergence and phylogenetic relationships among cyanobacteria isolated from reservoirs and well waters in the dry zone of Sri Lanka and also to identify possible toxin producers that promote potential health impacts to human and other water dependents. For that, reservoirs and well waters in dry zone were selected and isolated 45 cyanobacterial isolates and their level of diversity were assed to the classical five orders of cyanobacteria identified previously (Castenholz and Waterbury 1989). Further, attempted to determine the phylogenetic position of uncultured cyanobacterial isolates was also described.

## Methods

### Site selection, sampling and cyanobacterial isolation

**Fig. 1:**
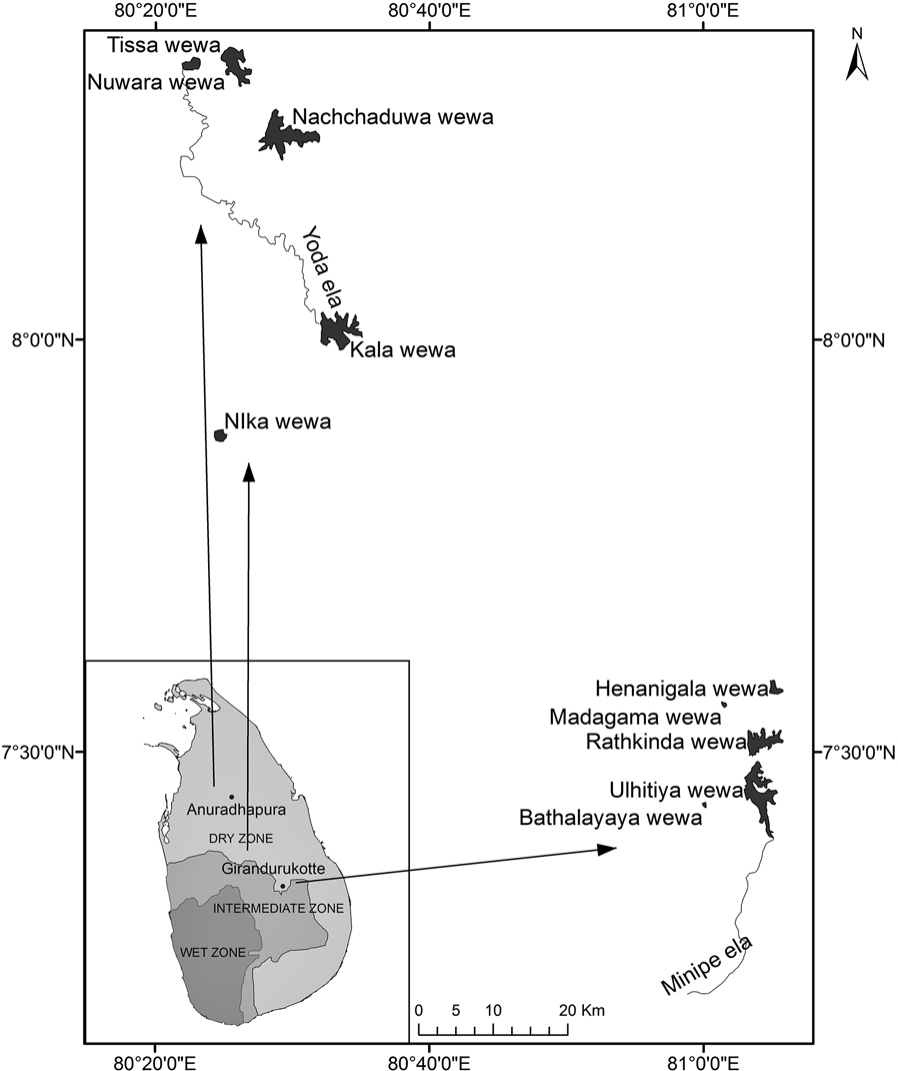
Sri Lankan map showing the reservoirs selected for samples collection in dry zone. Shaded regions show the climatic zones (dry, intermediate and wet) distribution

Water samples were collected from the dry zone of Sri Lanka (Fig. 1) including Tissa wewa reservoir (8°20′0″N, 80°22′0″E), Nuwara wewa reservoir (8°21′0″N, 80°25′0″E), Kala wewa reservoir (8°1′0″N, 80°31′0″E), Jaya Ganga reservoir (branch of Kala wewa), Nachchaduwa wewa reservoir (8°15′0″N and 80°28′60″E) in Anuradhapura District, Ulhitiya reservoir (7°27′26″N, 81°4′2″E), and Minipe Ela reservoir (7°12′36″N, 80°58′50″E) in Girandurukotte area, and Nika wewa reservoir (7°52′60″N, 80°25′0″E) in Kurunegala District. Sampling was carried out during 2010–2012 and sampling details are shown in Table 1. Hydrological and morphological characteristics for each sampling site was not considered except that these water bodies including the wells were man made and are being used for human consumption currently and as for the past decade. Criteria for selection was based on the fact that these selected reservoirs, and wells in selected districts belong to the dry zone of Sri Lanka and there are records of increasing number of human health impacts such as cancer and chronic kidney diseases in people living in these areas.

**Table 1:** Reservoirs selected for the study and their physiological characteristics

No.	Location/district	Coordinates	Sample code	Water source	Tem. (°C)	pH
1	Anuradhapura	Nuwara wewa 8°21′0″N, 80°25′0″E	Nuwara 1	Surface	33	8.48
2			Nu-M	Surface—Plankton net	33	8.48
3			AN 6	Bottom—1.5 m	28	8.60
4			AN 7	Bottom—11 m	28	8.43
5		Tissa wewa 8°20′0″N, 80°22′0″E	Tissa 1	Surface	30	8.71
6			AT2	Surface	28	8.34
7			AT3	Surface—Plankton net	28	8.35
8			AT4	Bottom—1.5 m	30	8.57
9		Jaya ganga	AJ2 W	Surface—Plankton net	31	8.02
10			AJJ2	Bottom—5 m	29	8.02
11		Kala wewa 8°1′0″N, 80°31′0″E	AKK1	Surface	29	8.45
12			AK2 W	Surface—Plankton net	32	8.68
13			AK 1	Bottom—15 m	27	8.48
14		Nachchaduwa wewa 8°15′0″N, 80°28′60″E	N-B/ASM1-A	Surface	31	8.48
15			N-B2/ASM1-A	Bottom—15 m	30	8.50
16	Girandurukotte (Badulla)	Henanigala Wewa 7°34′42″N, 81°3′49″E	GH1	Surface	28.5	7.76
17			GM2	Bottom—3 m	32	8.46
18			GH2	Soil	–	–
19		Ulhitiya Wewa 7°27′26″N, 81°4′2″E	GU	Surface	28	7.65
20			GUU1	Bottom—7 m	31	7.31
21			GUU2	Center—surface	31	7.84
22			GUU3	Surface	31	7.74
23		Ratkinda Wewa 7°30′49″N, 81°4′52″E	GR	Surface	28	7.51
24			GRR2	Surface	29	7.74
25			GRR1	Bottom—8.5 m	29	7.52
26		Minipe Ela 7°12′36″N, 80°58′50″E	GMMi2	Surface	29	7.66
27			GMR8	Center–Bottom—2 m	27	7.65
28			GMY6	Surface	29	7.49
29	Kurunegala	Nika wewa 7°52′60″N, 80°25′0″E	K/Nik	Surface	32	8.45
30			KN1	Bottom—7 m	31	8.48

Reservoirs’ water samples were collected into sterilized 2.5 L brown glass containers. Sampling was carried out from different sampling sites in duplicates. Both surface and bottom (using a hand corer- Wildco 2424-B) waters from different sampling sites were collected to represent the entire water body. Further, temperature and pH were recorded using a thermometer and a pH meter respectively. 25 shallow and deep dug wells used as the source of potable water were selected in random basis from Girandurukotte area and water samples were collected into 250 mL brown glass bottles in duplicates.

Collected water samples were concentrated by centrifugation (Beckman—CP Centrifuge) at 3500 rpm for 10 min and 500 μL of the resulting pellet and 500 μL from supernatant were inoculated into cyano specific BG11, BG11_0_, BG11_0_C and MLA liquid media. Cultures were incubated at room temperature (28 ± 2 °C) under fluorescent light (with intensity 4.8 × 10^−4^–5.9 × 10^−4^ cm^−2^ W) in 12–12 light dark cycling for about 3–4 weeks.

### DNA extraction, PCR amplification, DNA purification and sequencing

Both environmental and cultured samples were subjected for DNA extractions using Booms’ method (Boom et al. 1990). Polymerase Chain Reaction (PCR) was used to amplify 16S rRNA gene region (approximately 450 bp) to identify the presence of cyanobacteria using the modified protocols of Nübel et al. (1997). Cyanobacterial specific primers, forward primer; CYA359F (5′-GGGGAATCTTCCGCAATGGG-3′) along with the reverse primers; CYA781Rb (5′GACTACAGGGGTATCTAATCCCTTT-3′) and CYA781Ra (5′GACTACTGGGGTATCTA ATCCCTT-3′) for the identification of unicellular/non heterocyst filamentous and heterocyst forming cyanobacteria respectively. The resulted PCR products were electrophoresed in 1.5% agarose gels containing 10 µg/mL ethidium bromide and documented through a Gel Documentation system (Syngene, UK).

Amplified fragments were excised and purified with the genElute™ Gel Extraction Kit (SIGMA, USA) according to the manufacturer’s instructions. DNA sequencing was carried out at commercial facility by Macrogen Inc., South Korea. All the sequence data obtained were analyzed using DNA sequencing software programme BioEdit 7.0.9. To verify the cyanobacterial origin of sequenced samples, a BLAST (http://www.ncbi.nlm.nih.gov/BLAST/) was performed using the program “blastn” against the “Nucleotide collection (nr/nt)” database. PCR products that showed significant reproducibility after repeated (duplicate) analysis with CYA359F and CYA781Rb or CYA781Ra were selected for phylogenetic assessment.

### Phylogenetic analysis of isolated cyanobacterial strains using 16S rRNA gene

Phylogenetic tree was constructed for cyanobacterial 16S rRNA gene sequences of 45 isolates derived from this study (Table 2) and 26 toxic and non-toxic cyanobacterial sequences from GenBank at NCBI database (Additional file 1: Table S1) in the view of elucidating the taxonomic positions of cyanobacteria belonging to five orders and unclassified cyanobacterial isolates, and their toxicity. *E. coli* strain (X80721) was used as an out group to root the tree. DNA sequences were aligned and compared with sequence data available in the NCBI database using the ClustalW alignment algorithm. All positions containing gaps and missing data were eliminated from the dataset. A neighbour joining (NJ) max-mini branch-and-bound analysis using MEGA 4.0 ANALYSIS software (Tamura et al. 2004) was used to illustrate the relationship of partial 16S rRNA gene of representative cyanobacteria. Bootstrap analyses were performed with 100 replicates and only bootstrap percentages above 50 were shown at the branch nodes of phylogenetic distance trees.

**Table 2:** 16S rRNA gene sequences of Cyanobacteria (derived from this study) selected for phylogenetic analysis

No.	Sample code	Isolated reservoir/well	Isolate	Genebank accession no.
1	GH2	Henanigala	*Phormidium* sp. enrichment culture clone GH2	HM640024
2	GH2	Henanigala	*Phormidium animale* GK12	KF321927
3	GU	Ulhitiya	Uncultured Oscillatoria sp.GK13	KF321928
4	AJ2 W	Jaya ganga	*Raphidiopsis curvata* AJ1	KF321929
5	AN2W2	Nuwara wewa	*Cylindrospermopsis raciborskii* AN3	KF321930
6	T4	Tissa wewa	Uncultured cyanobacterium AT4	KF321931
7	T3	Tissa wewa	Uncultured cyanobacterium AT3	KF321932
8	T2	Tissa wewa	Uncultured cyanobacterium AT2	KF321933
9	GU	Ulhitiya	Uncultured *Synechococcus* sp. GK14	KF321934
10	GR	Ratkinda	Uncultured cyanobacterium GK15	KF321935
11	K/Nik	Nika wewa	Uncultured Chroococcales cyanobacterium KN1	KF321936
12	Tissa 1	Tissa wewa	Uncultured cyanobacterium AT5	KF321937
13	Nuwara 1	Nuwara wewa	Uncultured cyanobacterium AN2	KF321938
14	T2	Tissa wewa	Uncultured cyanobacterium AT6	KF321939
15	GMMi2	Minipe ela	Uncultured cyanobacterium GK1	KF321940
16	G/63-A	Well water	Uncultured cyanobacterium GK2	KF321941
17	G/147-A	Well water	Uncultured *Leptolyngbya* sp.GK16	KF321942
18	G/111-A	Well water	Uncultured cyanobacterium GK3	KF321943
19	G/100-A	Well water	Uncultured cyanobacterium GK4	KF321944
20	G/3-A	Well water	Uncultured cyanobacterium GK5	KF321945
21	G/60	Well water	Uncultured cyanobacterium GK6	KF321946
22	G/148-A	Well water	Uncultured cyanobacterium GK7	KF321947
23	T-3/MLA-B	Thuruvila wewa	Unicellular cyanobacterium A1	KF 359768
24	Nu-M/MLA-A	Nuwara wewa	*Radiocystis* sp.A2	KF 359770
25	N-B/ASM1-A	Nachchaduwa	Unicellular cyanobacterium A3	KF 359771
26	AT3-MLA	Tissa wewa	Prochlorales cyanobacterium HM17	KF 321965
27	G/100-C-BG11_0_C	Well water	Filamentous cyanobacterium HM15	KF 321963
28	I/23-1-BG11_0_	Well water	*Hapalosiphon welwitschii* HM14	KF 321962
29	AT2-MLA	Tissa wewa	*Leptolyngbya* sp. HM13	KF 321961
30	G/81-BG11_0_	Well water	Nostocales cyanobacterium HM12	KF 321960
31	I/23-1-BG11	Well water	*Hapalosiphon welwitschii* HM11	KF 321959
32	G/79-C-BG11	Well water	*Phormidium* sp. HM10	KF 321958
33	G/146-D-MLA	Well water	*Mastigocladus* sp. HM9	KF 321957
34	G/84-D-BG11_0_	Well water	*Anabaena sphaerica* HM8	KF 321956
35	G/66-BG11_0_	Well water	*Nostoc punctiforme* HM7	KF 321955
36	GMY6-BG11	Minipe ela	Chroococcales cyanobacterium HM6	KF 321954
37	G-127-MLA	Well water	*Mastigocladus* sp. HM5	KF 321953
38	G/100-D-BG11_0_C	Well water	Unicellular cyanobacterium HM4	KF 321952
39	G/183-44-BG11_0_	Well water	*Tolypothrix* sp. HM3	KF 321951
40	GMR8–BG11	Minipe ela	*Chroococcidiopsis* sp. HMI	KF 321949
41	I/23-1-MLA	Well water	*Chroococcus* sp. HM2	KF 321950
42	G/100-A-BG11_0_C	Well water	Filamentous cyanobacterium HM16	KF 321964
43	G/5-A-BG11	Well water	Chroococcales cyanobacterium GK8	KF 359772
44	G/3-A-MLA	Well water	*Leptolyngbya* sp. GK10	KF 359774
45	G/169-A-BG11	Well water	*Synechococcus* sp. GK9	KF 359773

## Results

### DNA extraction, PCR amplification, DNA purification and sequencing

All the samples showed positive amplification for 16S rRNA either with CYA781Rb or CYA781Ra reverse primers indicating the presence of unicellular/non heterocyst filamentous and heterocyst forming filamentous cyanobacteria (Additional File 1: Fig. S1). All 45 sequenced cyanobacterial isolates were deposited in GenBank at National Center for Biotechnology Information (NCBI) database with sample details.

Among 45 isolates, 20 isolates were classified as unidentified cyanobacteria since they classified into unculturable and/or either to unicellular or filamentous cyanobacteria. Although those 20 isolates showed 96–99% sequence similarity to previously record unculturable and/or either to unicellular or filamentous cyanobacteria recorded in NCBI database, with 16S rRNA gene sequences, these isolates could not be assigned to any taxonomic level. Therefore, these cyanobacterial isolates could be considered as novel cyanobacterial genera. Further, among 25 identified isolates, seven isolates were belonged to order Chroococcales, one to order Pleurocapsales, seven to order Oscillatoriales, six isolates to order Nostocales and four isolates to the order Stigonematales. Of 25 identified isolates, seven isolates (KF321927, KF321929, KF321930, KF 321962, KF 321959, KF 321956, KF 321955) were identified up to species level. Those isolates showed 98–100% showed similarity to previously reported cyanobacterial sequences in NCBI database. Based on these results, order Chroococcales, Oscillatoriales, and Nostocales cyanobacteria could be considered as the versatile genus since they were widely distributed in the studied area. Further, based on 16S rRNA results obtained from the present study clearly recognized that there is a significant diversity in cyanobacteria within the dry zone in the country.

### Phylogenetic analysis of isolated cyanobacterial strains using 16S rRNA gene

**Fig. 2:**
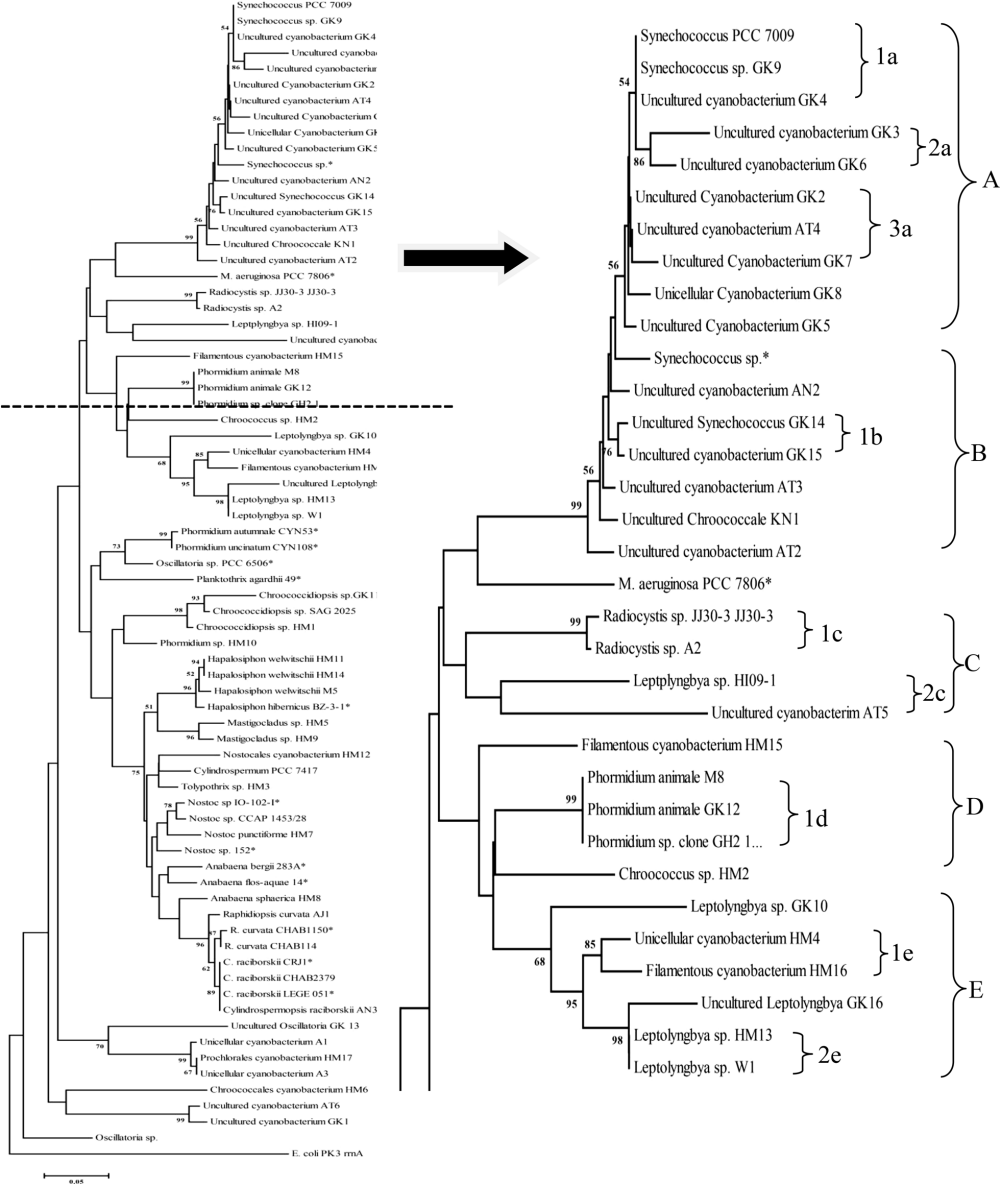
Phylogenetic tree for cyanobacterial 16S rRNA gene sequences constructed using Neighbour Joining (NJ) method. The *scale bar* represents five base substitutions for 100 nucleotide positions. Bootstrap percentages above 50% calculated from 100 re-sampling are indicated at the nodes

**Fig. 3:**
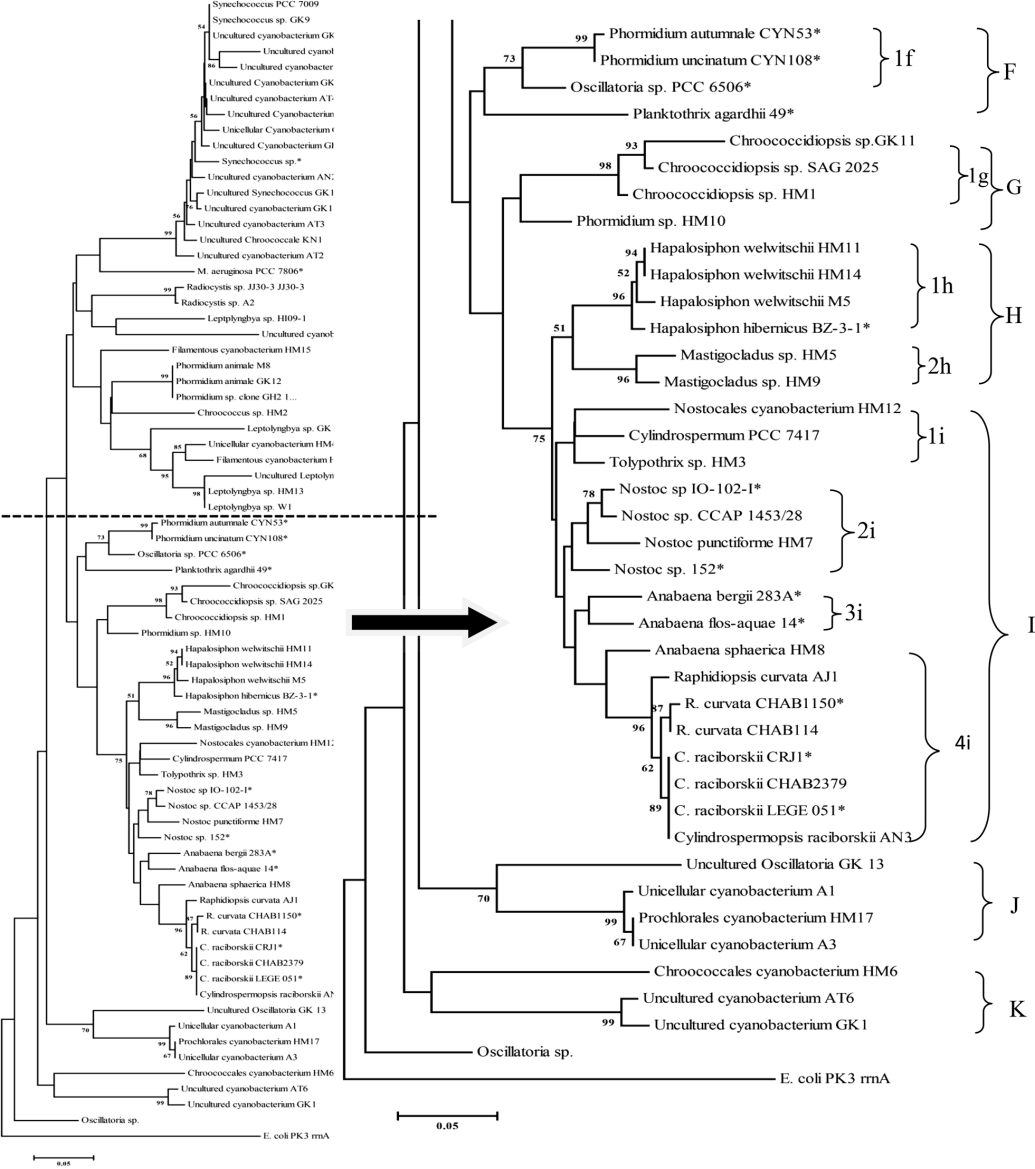
Phylogenetic tree for cyanobacterial 16S rRNA gene sequences constructed using Neighbour Joining (NJ) method. The *scale bar* represents five base substitutions for 100 nucleotide positions. Bootstrap percentages above 50% calculated from 100 re-sampling are indicated at the nodes

The phylogenetic tree (Figs. 2, 3), clearly demonstrated eleven main clusters (A–K) irrespective to their toxicity. Clustered pattern showed a good lineage to major taxonomic order levels in cyanobacteria, Chroococcales (cluster A, B, C, J and K), Pleurocapsales (cluster G), Oscillatoriales (cluster D, E and F), Nostocales (cluster I) and Stigonematales (cluster H) with few deviations.

Cluster A, showed two sub clusters; 1a and 2a. In cluster 1a, *Synechococcus* GK9 and uncultured cyanobacterium GK4 clustered with non toxic *Synechococcus* PCC 7009 sharing 100% sequence similarity with 54% bootstrap (BT) support while uncultured cyanobacterium GK3 and GK6 clustered together sharing >96% sequence similarity with 86% BT support. Cluster 2a contained uncultured cyanobacterium GK2, AT4 and GK7 sharing >98% similarity. Other uncultured cyanobacterium GK8 and GK5 clustered with 2a sharing >96% similarity and 56% BT support. Uncultured cyanobacterium GK3 and GK6 deviated from 1% and similarly uncultured cyanobacterium GK5 and unicellular cyanobacterium GK8 deviated from 1%. However all four sequences were clustered with 1a and 2a sub clusters sharing >98% similarity and 56% BT support. Therefore, Cluster A comprised non-toxic unicellular cyanobacterial strains sharing >96% similarity.

Considering Cluster B, it consisted of a single sub cluster; 1b which composed uncultured *Synechococcus* GK14 and uncultured cyanobacterium GK15 which showed >98% similarity and clustered with 56% BT support. Further, sub cluster 1b along with uncultured cyanobacterium AN2 and AT3 clustered with toxic *Synechococcus* sp. sharing >98% similarity and 56% BT support. Further, 1b clustered with uncultured cyanobacterium AT3 and KN1 grouping the cluster B. Furthermore, uncultured cyanobacterium AT2 clustered with 1b with 99% BT support. Therefore cluster B contained toxic unicellular cyanobacterial strains sharing >98% similarity. Further, both cluster A and cluster B re-clustered with toxic *M. aeruginosa* PCC 7806 sharing >94% similarity.

Cluster C contained two sub clusters; sub cluster 1c and 2c. Considering cluster 1c, *Radiocystis* sp. A2 derived from this study clustered with *Radiocystis* sp. JJ30-3 from NCBI database with 99% similarity and with significant 99% BT support. Similarly in sub cluster 2c, uncultured cyanobacterium AT5 isolate clustered with non-toxic *Leptolyngbya* sp. H109-1 and showed lowest similarity sharing 90% similarity. Further the low BT value at the nodes within the cluster was consistent with this result. Sub cluster 1c and 2c deviated from 5% and also sub cluster 1c and 2c deviated from toxic *M. aeruginosa* PCC 7806 and clusters A and B from 2%.

Cluster D composed a distinct cluster; 1d and a separate single branch of *Chroococcus* sp. HM2. *Phormidium animale* GK12 and *Phormidium* sp. clone GH2 derived from this study clustered with *Phormidium animale* M8 from NCBI database with 100% similarity and 99% BT support.

Considering cluster E, it composed two distinct sub clusters; 1e and 2e with 95% BT support and a separate single branch of *Leptolyngbya* sp. GK10. 1e consisted uncultured cyanobacterium HM4 and filamentous cyanobacterium HM16 sharing >97% similarity and 85% BT support while sub cluster 2e further sub divided into two branches comprising three *Leptolyngbya* sp. GK16, HM13 and W1 with 98% BT support. *Leptolyngbya* sp. HM13 derived from this study clustered with non-toxic *Leptolyngbya* sp. W1 sharing 100% similarity and both were deviated from uncultured *Leptolyngbya* sp. GK16 from 4%. Two sub clusters cluster with *Leptolyngbya* sp. GK10 with >96% similarity and 68% BT support. Therefore, cluster E contained non-toxic filamentous cyanobacterial isolates sharing >94% similarity. Further, cluster D and E re-clustered with filamentous cyanobacterium HM15, sharing >93% sequence similarity.

Cluster F consisted of a single sub cluster 1f and two distinctly separated branches. Complete cluster F comprised toxic Oscillatoriales species; *Phormidium autumnale* CYN53, *Phormidium unicinatum* CYN108, *Oscillatoria* sp. PCC 6506 and *Planktothrix agardhii* 49 which were from NCBI database sharing >92% similarity.

Considering cluster G, it composed a single sub cluster 1g and a single separate branch *Phormidium* sp. HM10. *Chroococcidiopsis* sp. GK11 and HM1 grouped with *Chroococcidiopsis* sp. SAG 2025 completing sub cluster 1g with 98% BT support. *Chroococcidiopsis* sp. HM1 grouped with *Chroococcidiopsis* sp. SAG 2025 sharing >96% similarity and 93% BT support.

Cluster H composed two lineages; 1h and 2h with 51% BT support. Sub cluster 1h composed four *Hapalosiphon* isolates while 2 h composed two *Mastigocladus* isolates. In sub cluster 1h, *Hapalosiphon welwitschii* HM11 and HM14 were identical and clustered together with 100% sequence similarity and 94% BT support while both re-clustered with non-toxic *Hapalosiphon welwitschii* M5 and with toxic *Hapalosiphon hibernicus* BZ-3-1 isolate both with 99% sequence similarity. Both branches were supported with 52 and 96% BT support respectively. Considering sub cluster 2 h, *Mastigocladus* sp. HM5 and HM9 clustered together sharing 99% similarity. Both sub clusters 1h and 2h re-clustered with 51% BT support.

Considering cluster I, the whole cluster composed of Nostocales cyanobacterial isolates with four distinct clusters; 1I, 2I, 3I and 4I. Sub cluster 1I composed three isolates Nostocales cyanobacterium HM12 and *Tolypothrix* sp. HM3 derived from this study and non-toxic *Cylindrospermum* PCC 7417from NCBI database sharing >94% sequence similarity. Sub cluster 2I composed two isolates; toxic Nostoc sp.IO-102-I and non-toxic CCAP 1453/28 from NCBI database sharing 99% sequence similarity and separate two clusters; *Nostoc* strain 152 and *Nostoc punctiforme* from NCBI database sharing >96% sequence similarity. Further, sub cluster 3I composed two toxic *Anabaena* strains which are known to produce CYN; Anabaena *bergii* 283 and *Anabaena flos*-*aquae* 14 from NCBI data base sharing over 96% sequence similarity. Considering sub cluster 4I, it composed two divisions; first division consisting three *Raphidiopsis* species and the other with four *Cylindrospermopsis* species with 62% BT support along with a separate branch of *Anabaena sphaerica* HM8. *Raphidiopsis* division consisted of two closely related toxic and non-toxic strains; CHAB1150 and CHAB 114 from NCBI database with 87% BT support along with *Raphidiopsis curvata* AJ1 derieved from this study with 96% BT support. Two toxic and non-toxic strains were deviated from *Raphidiopsis curvata* AJ1 by 1%. Further, *Cylindrospermopsis* division composed; non-toxic *C. raciborskii* CRJ1, *C. raciborskii* CHAB 2379 and toxic *C. raciborskii* LEGE 051 from NCBI database along with *C. raciborskii* AN3 derived from this study sharing 100% sequence similarity and 89% BT support.

Cluster J consisted of a single sub cluster 1J along with a separate branch of Uncultured Oscillatoria GK13 with 70% BT support. Sub cluster 1J composed Prochlorales cyanobacterium HM17 and unicellular cyanobacterium A3 sharing 100% sequence similarity while clustered with unicellular cyanobacterium A3 sharing over 99% sequence similarity with 67 and 99% BT support respectively.

Considering cluster K, it composed a sub cluster 1K along with a separate branch of Chroococcales cyanobacterium HM6. Sub cluster 1K clustered uncultured cyanobacterium AT6 and GK1 sharing >98% sequence similarity with 99% BT support.

## Discussion

According to the climatic distribution in the country, the Anuradhapura, Kurunegala and Girandurukotte area of Badulla districts belong to the dry zone of Sri Lanka. These localities have a number of reservoirs which are used for drinking, irrigation and other recreational activities and are also of an aesthetic value.

Assigning uncultured cyanobacteria into particular genera either by morphology or biochemically and understanding their toxicity is a difficult task. Thus the study utilized molecular tools to overcome the above constrains. The phylogenetic relationship arose from 16S rRNA gene sequence comparison supported the traditional classification of cyanobacteria which was based on morphological characters. All eleven clusters clearly demonstrated five cyanobacterial orders with few exceptions and therefore it showed the suitability of 16S rRNA gene for taxonomic differentiation. This was also supported in the work done by Nelissen et al. (1992), Li et al. (2001), Wilmotte and Herdman (2001). According to the results, out of 45 isolates, 24 uncultured cyanobacteria were able to place on their taxonomic positions up to order level with 16S rRNA sequences. Among them, 13 isolates were positioned in cluster A which composed of Chroococcales cyanobacteria and further to *Synechococcus* cyanobacteria. Uncultured cyanobacterium AN2, GK14, GK15, and AT3 gathered with toxic *Synechococcus* sp. with >98% genetic similarity while sub cluster 1a, 2a and 3a clustered with toxic *M. aeruginosa* PCC 7806 (Tillett et al. 2001) with >90% sequence similarity. Therefore, Nuwara wewa (AN2), Tissa wewa (AT3, AT2 and AT4), Nika wewa (KN1) and well waters in Girandurukotte (GK9, GK3, GK6, GK2, GK7, GK8, GK5, GK14, and GK15) contained potential MC producing cyanobacterial strains which belonged to order Chroococcales.

Though *Radiocystis* species were also known to produce MC (Vieira et al. 2003; Lombardo et al. 2006), no sequence of MC producing *Radiocystis* strain has been deposited in the NCBI database and therefore toxin producing ability of *Radiocystis* could not be determined. However, *Radiocystis* sp. A2 formed a tight cluster (99% similarity) with *Radiocystis* sp. JJ30-3 in which the toxicity was unknown. Therefore, *Radiocystis* sp. A2 which was isolated from a Nuwara wewa reservoir may or may not have the genetic potential to produce MC. Similarly, *Chroococcidiopsis* sp. SAG 2025 in which the toxicity unknown clustered with GK11 and HM1 with high BT support (98%). Further, *Leptolyngbya, Phormidium* and *Oscillatoria* were polyphyletic and this polyphyletic nature of order Oscillatoriales was also reported previously by other studies (Nelissen et al. 1992; Garcia-Pichel et al. 2001). Similarly, Chroococcales cyanobacterial isolates (in cluster A, J and K) also reflected polyphyletic nature which was also been reported by the previous authors (Nelissen et al. 1992; Garcia-Pichel et al. 2001).

*Hapalosiphon* sp. BZ-3-1 was known to produce MC (Prinsep et al. 1992) and therefore *Hapalosiphon* sp. HM11 and HM14 have a potential to produce MC since they shared >97% similarity with 94% BT support. Those *Hapalosiphon* isolates HM11 and HM14 were isolated from well waters. Interestingly, cluster I clustered all Nostocales cyanobacteria reflected a monophyletic behaviour. The monophyletic nature of the heterocysts forming cyanobacteria in order Nostocales was reported previously by Nelissen et al. (1992), Wilmotte and Herdman (2001) and Lyra et al. (2001). Considering cluster 2i, *Nostoc* sp. HM7 clustered with highly toxic MC producing *Nostoc* sp. IO-102-I (Oksanen et al. 2004) and hepatotoxic *Nostoc* sp. 152 (Lyra et al. 2001) sharing >97% sequence similarity and also with non-toxic *Nostoc* sp. CCAP 1453/28 sharing >97% similarity. Therefore, *Nostoc* sp. HM7 isolated from a well water in Girandurukotte may or may not have the potential to produce hepatotoxins. *Raphidiopsis curvata* AJ1 isolate (in cluster 4i) which was isolated from Jaya ganga reservoir, clustered with *Raphidiopsis curvata* CHAB1150 which is a CYN producer (Jiang et al. 2012) sharing >98% similarity and therefore *Raphidiopsis curvata* AJ1 has a potential to produce CYN. Moreover, all *C. raciborskii* spp. irrespective to their toxicity formed a tight cluster sharing 100% sequence similarity with 89% significant BT support. Among the species, *C. raciborskii* LEGE 051 was known to produce CYN (Moreira et al. 2011) and therefore *C. raciborskii* AN3 isolated from Nuwara wewa may or may not produce CYN. The high similarity of DNA sequences of *Raphidiopsis* and *Cylindrospermopsis* highlighted that the strains of these two genera were closely and phylogenetically related supporting the conclusions drawn by Stucken et al. (2010) and Wu et al. (2011). However, the isolates of *Raphidiopsis* and *Cylindrospermopsis* distributed randomly in the cluster I irrespective to their toxicity, suggesting that no clear correspondence was shown between toxicity and phylogeny. This was also supportive with the cluster 2i where both highly toxic and non-toxic *Nostoc* sp. IO-102-I and *Nostoc* sp. CCAP 1453/28 clustered sharing 100% similarity.

According to the results obtained in this study, it was evident that 16S rRNA gene is an effective tool in construing phylogenetic relationships between different genera within order level. This phenomenon was also supportive with the previous studies done by several authors. Also, the 16S rRNA gene has been useful in identifying and classifying strains that belong to a single clade (Palinska et al. 1996; Otsuka et al. 1999). For examples, a study done by Nelissen et al. (1992) using 16S rRNA sequences of five strains of *Pseudanabaena,* concluded that they were nearly identical, and hence confirmed them as a single monophyletic taxon. Further, a phylogeny study by Honda et al. 1999 using 16S rRNA sequence data, clustered members of the genus *Synechococcus* and concluded that the strains were closely related.

Though 16S rRNA sequence analysis is relatively useful in determining evolutionary relationships among organisms at the genus level; it is not that helpful in differentiating at species or sub species levels due to the fact that 16S molecules is almost constant (with the total variation of about 200 bp for a mean length of 1500 bp (Rainey et al. 1996; Moreira and Philippe 2010) and thus different genes cannot be easily separated by size. Additionally, even though the 16S rDNA sequence consists of hyper variable and extremely informative regions for close relationships, it is often not divergent adequately to give good separation in close relations (Fox et al. 1992; Normand et al. 1996; Turner 1997). Further, in comparison to most of the protein-encoding genes, with time this sequence is more susceptible to changes. This is due to the fact that protein-encoding genes are more tolerable to variations in amino acid codon sequences (silent mutations in the third nucleotide in a codon) than rRNA genes. Moreover, the level of degeneracy in the 16S rRNA gene is much less than in protein-coding genes (Vinuesa et al. 1998). Due to its conservative nature, the 16S rRNA gene sequence is lacking the needed divergence for comparing species within generic clades (Bolch et al. 1999).

According to the phylogeny analysis, reservoirs and well waters in Sri Lanka consisted rich cyanobacterial diversity which also have a potential to produce cyanotoxins. Since 16S rRNA phylogeny failed to reflect a strong image on toxicity in isolated strains, it is suitable to use multi-gene analysis for a better resolution (Tanabe et al. 2007; Wu et al. 2011).

## Conclusion

Based on 16S rRNA results obtained from the present study, it was clearly acknowledged the significant diversity in cyanobacteria with potential microcystin (MC) and cylindrospermopsin (CYN) producers within the dry zone in the country. Among 45 isolates, 20 isolates were classified as unidentified cyanobacteria and considered as novel cyanobacterial genera. Among 25 identified isolates, seven isolates were belonging to order Chroococcales, one to order Pleurocapsales, seven to order Oscillatoriales, six isolates to order Nostocales and four isolates to the order Stigonematales. Of 25 identified isolates, seven isolates were identified up to species level.

With 16S rRNA Phylogenetic results, eleven clusters clearly demonstrated five cyanobacterial orders with more than 90% similarity irrespective to their toxicity which showed the suitability of 16S rRNA gene for taxonomic differentiation. Sixteen isolates had the potential to produce MC and two isolates to produce CYN. Twenty uncultured cyanobacterial isolates were able to place on their taxonomic positions up to order level. Findings of the study confirm the rich cyanobacterial diversity and the divergence among the potential cyanotoxin producers in the dry zone water bodies and is an indication regarding the numerous health impacts faced by the people living in the dry zone of Sri Lanka.

Therefore, the study underlines a need of continuous monitoring programmes, the efficient management of water bodies and correct toxicity assessment by accurate identification of problematic cyanobacterial species is necessary for the waters used as sources of drinking water.

## Additional file

**Additional file 1.** **Table S1**. Cyanobacterial 16S rRNA gene sequences (from NCBI database) selected for phylogenetic analysis. **Fig S1**. Photograph of an ethidium-bromide stained gel showing the purified PC products of 16S rRNA partial sequences of six cyanobacterial isolates.
