# Supplementary material for: Genetic divergence among toxic and non-toxic cyanobacteria of the dry zone of Sri Lanka
Source: Springerplus. 2016 Nov 28;5(1):2026. doi: 10.1186/s40064-016-3680-5 (PMC5125326; doi:10.1186/s40064-016-3680-5)
Supplement: Supplementary file 1 — Additional file 1. Table S1. Cyanobacterial 16S rRNA gene sequences (from NCBI database) selected for phylogenetic analysis. Fig S1. Photograph of an ethidium-bromide stained gel showing the purified PC products of 16S rRNA partial sequences of six cyanobacterial isolates. [file 40064_2016_3680_MOESM1_ESM.docx]

**Additional File 1**

**Table S1. Cyanobacterial 16S rRNA gene sequences (from NCBI database) selected for phylogenetic analysis**

| **No.** | **Isolate** | **Genebank accession No.** | **Toxicity** |
| --- | --- | --- | --- |
| 1 | *Microcystis aeruginosa* strain PCC 7806 | AF139299 | Toxic |
| 2 | *Raphidiopsis curvata* CHAB1150 | JN873923 | Toxic |
| 3 | *Raphidiopsis curvata* CHAB114 | FJ890621 | Non toxic |
| 4 | *Cylindrospermopsis raciborskii* CJR1 | AB115485 | Non toxic |
| 5 | *Cylindrospermopsis raciborskii* LEGE 051 | HQ407326 | Toxic |
| 6 | *Cylindrospermopsis raciborskii* CHAB2379 | FJ890634 | Non toxic |
| 7 | *Oscillatoria sp*. PCC 6506 | AY768397 | Toxic |
| 8 | *Oscillatoria sp*. | AJ133106 | Non toxic |
| 9 | *Phormidium autumnale* CYN53 | JX088083 | Toxic |
| 10 | *Phormidium animale* M8 | KC768847 | Non toxic |
| 11 | *Anabaena bergii* | AF160256 | Toxic |
| 12 | *Anabaena Flos-aquae* 14 | AJ133152 | Toxic |
| 13 | *Cylindrospermum* sp. PCC 7417 | AJ133163 | Non toxic |
| 14 | *Nostoc* sp. IO-102-I | AY566855 | Toxic |
| 15 | *Phormidium* cf. *uncinatum* CYN108 | JX088078 | Toxic |
| 16 | *Nostoc* sp. 152 | AJ133161 | Toxic |
| 17 | *Nostoc* sp. CCAP 1453/28 | HF678493 | Non toxic |
| 18 | *Hapalosiphon hibernicus* BZ-3-1 | EU151900 | Toxic |
| 19 | *Hapalosiphon welwitschii* M5 | KC768846 | Non toxic |
| 20 | *Radiocystis* sp. JJ30-3 | AM710389. | Non toxic |
| 21 | *Chroococcidiopsis* sp. CCMP1489 | AJ344556 | Non toxic |
| 22 | *Planktothrix agardhii* | AJ133167 | Toxic |
| 23 | *Synechococcus* sp. clone K1-09 | GU784980v | Toxic |
| 24 | *Synechococcus* PCC7009 | AF216945 | Non toxic |
| 25 | *Leptolyngbya* sp. HI09-1 | GU111930 | Non toxic |
| 26 | *Leptolyngbya* sp. W1 | GU967417 | Non toxic |
| 27 | *E. coli* | X80721 | Unknown |

**1 2 3 4 5 6 7 8**

**
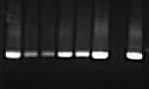
**

**~ 450 bp**

**Fig S1. Photograph of an ethidium-bromide stained gel showing the purified PC products of 16S rRNA partial sequences of six cyanobacterial isolates.**

Lane 1: G/100-C-BG11_0_C; Lane 2: I/23-1-BG11; Lane 3: G/81-BG11_0_; Lane 4: G/84-D; Lane

5: I/23-1 – BG11_0_; Lane 6- G/66- BG11_0;_ Lane 7: negative control (water); Lane 8: positive control (*Microcystis aeruginosa* strain PCC 7941).
